# Successful application of airway pressure release ventilation in a child with severe acute respiratory distress syndrome induced by trauma: a case report

**DOI:** 10.1186/s12890-024-02894-1

**Published:** 2024-02-12

**Authors:** Jing Su, Xin Tie, Yao Chen, Tongjuan Zou, Wanhong Yin

**Affiliations:** Department of Critical Care Medicine, West China Hospital, Sichuan University, Chengdu, 610041 China

**Keywords:** Acute respiratory distress syndrome, Airway pressure release ventilation, Trauma, Case report

## Abstract

**Background:**

Trauma has been identified as one of the risk factors for acute respiratory distress syndrome. Respiratory support can be further complicated by comorbidities of trauma such as primary or secondary lung injury. Conventional ventilation strategies may not be suitable for all trauma-related acute respiratory distress syndrome. Airway pressure release ventilation has emerged as a potential rescue method for patients with acute respiratory distress syndrome and hypoxemia refractory to conventional mechanical ventilation. However, there is a lack of research on the use of airway pressure release ventilation in children with trauma-related acute respiratory distress syndrome. We report a case of airway pressure release ventilation applied to a child with falling injury, severe acute respiratory distress syndrome, hemorrhagic shock, and bilateral hemopneumothorax. We hope this case report presents a potential option for trauma-related acute respiratory distress syndrome and serves as a basis for future research.

**Case presentation:**

A 15-year-old female with falling injury who developed severe acute respiratory distress syndrome, hemorrhagic shock, and bilateral hemopneumothorax was admitted to the surgical intensive care unit. She presented refractory hypoxemia despite the treatment of conventional ventilation with deep analgesia, sedation, and muscular relaxation. Lung recruitment was ineffective and prone positioning was contraindicated. Her oxygenation significantly improved after the use of airway pressure release ventilation. She was eventually extubated after 12 days of admission and discharged after 42 days of hospitalization.

**Conclusion:**

Airway pressure release ventilation may be considered early in the management of trauma patients with severe acute respiratory distress syndrome when prone position ventilation cannot be performed and refractory hypoxemia persists despite conventional ventilation and lung recruitment maneuvers.

## Introduction

Acute respiratory distress syndrome (ARDS) is a heterogeneous clinical syndrome with high mortality despite advances in supportive therapy. The incidence of ARDS was 10.4% of ICU admissions with hospital mortality of 46.1% for most severe cases [1]. Trauma-related ARDS accounts for approximately 4–7% of ARDS [1]. Managing respiratory support in patients with trauma-related ARDS is often challenging due to the presence of various comorbidities such as thoracic injury, pulmonary contusion, fat embolism syndrome, transfusion-related lung injury, neurogenic pulmonary edema, and elevated intracranial pressure [2]. Conventional ventilation modes may fail to improve hypoxemia in these patients while implementing lung-protective ventilation strategies [3]. Management for pediatric ARDS gets more complicated as children represent a diverse and heterogeneous population in terms of age, weight, diseases, and respiratory mechanics. The Second Pediatric Acute Lung Injury Consensus Conference (PALICC-2) guidelines have not yet recommended a preferred ventilation mode [4].

Airway pressure release ventilation (APRV) is a mode of ventilation typically viewed as a rescue or alternative mode for patients with ARDS and hypoxemia refractory to conventional mechanical ventilation. Presumed advantages of APRV include lung-protective recruitment, stabilization of open lung, reduced repetitive alveolar collapse with shearing, unrestricted spontaneous breathing, hemodynamic improvement, and reduced paralysis and sedation requirement [5]. The efficacy and safety of APRV in children with trauma-related ARDS remain unclear. In this case study, we report the use of APRV in a child with falling injury, severe ARDS and bilateral hemopneumothorax. The patient suffered refractory hypoxemia despite the treatment of conventional mechanical ventilation, deep analgesia and sedation, muscle relaxation, and PEEP titration for recruitment. The patient was unable to perform prone positioning ventilation due to multiple fractures. After the use of APRV, the patient’s oxygenation improved significantly without hemodynamic compromise.

## Case presentation

**Fig. 1:**
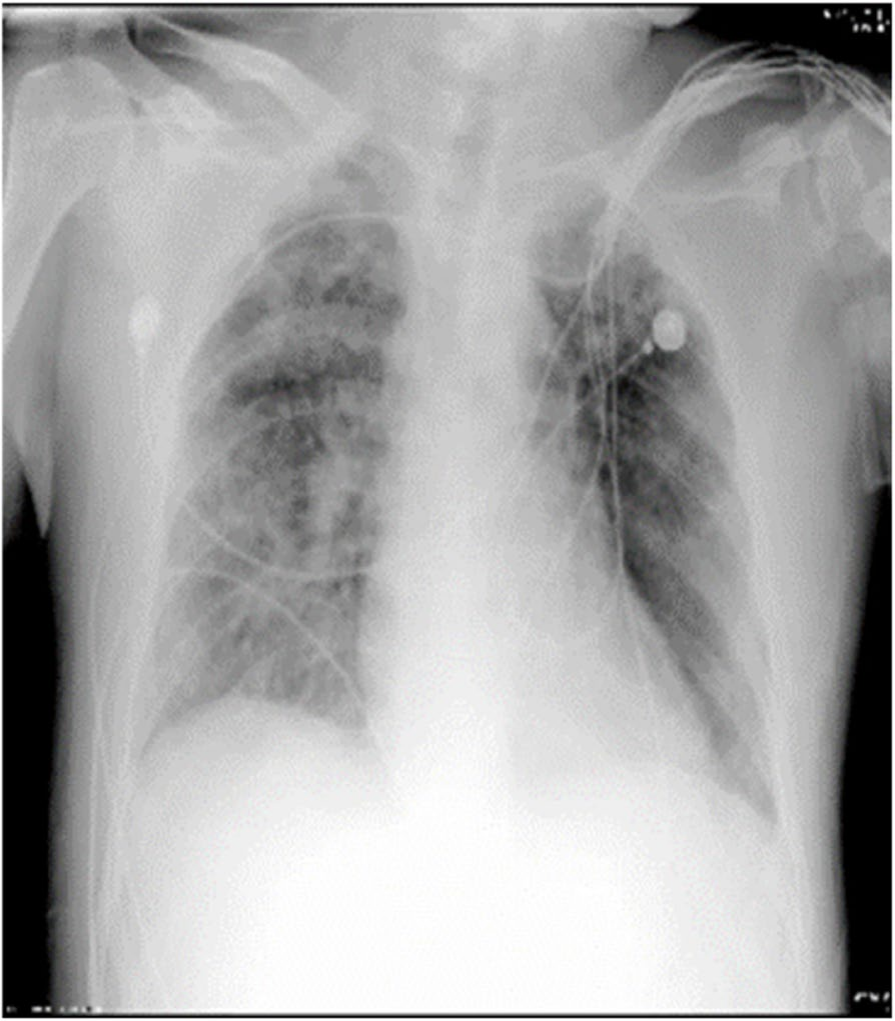
Chest X-ray on admission

**Fig. 2:**
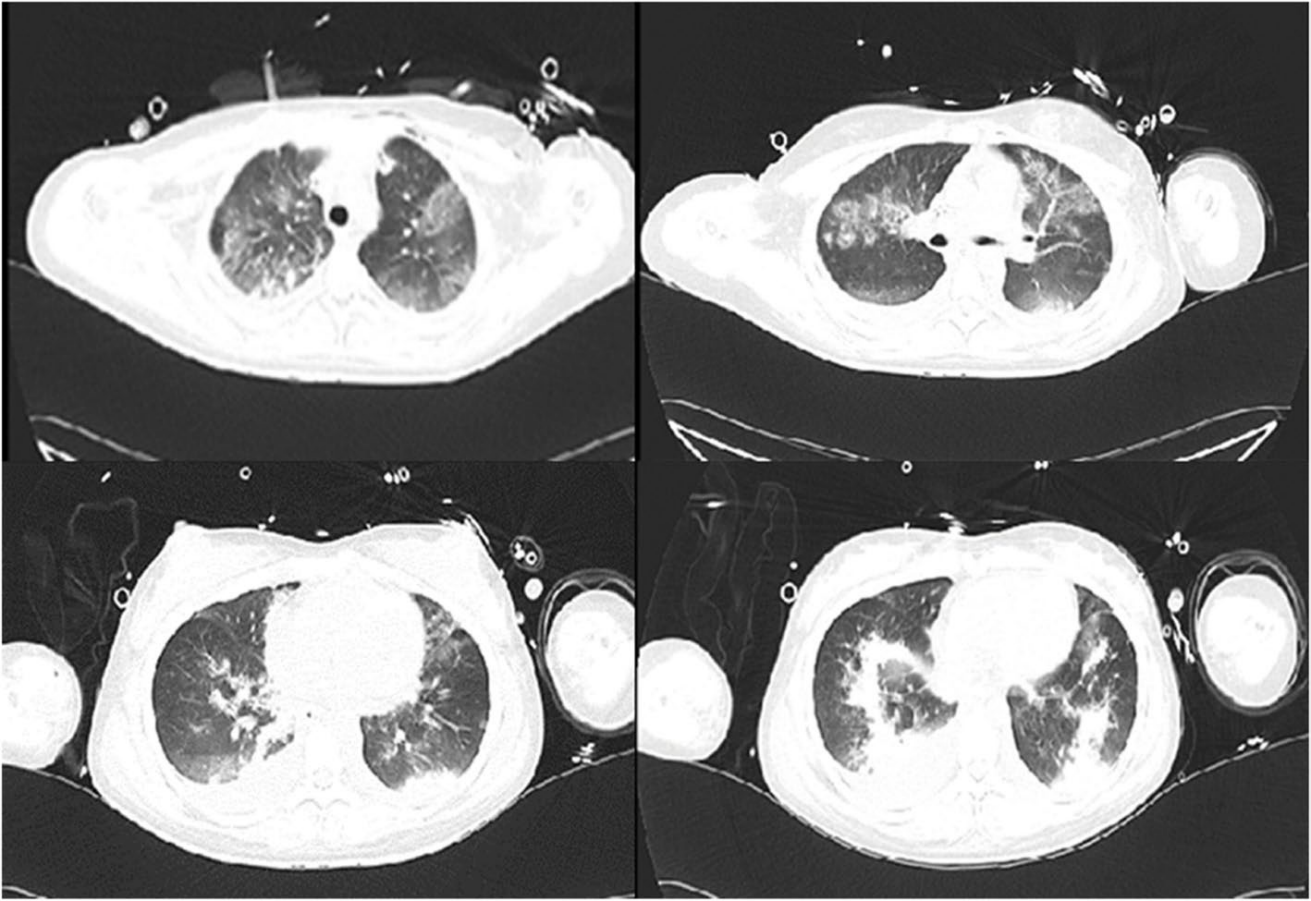
Chest CT on admission

**Fig. 3:**
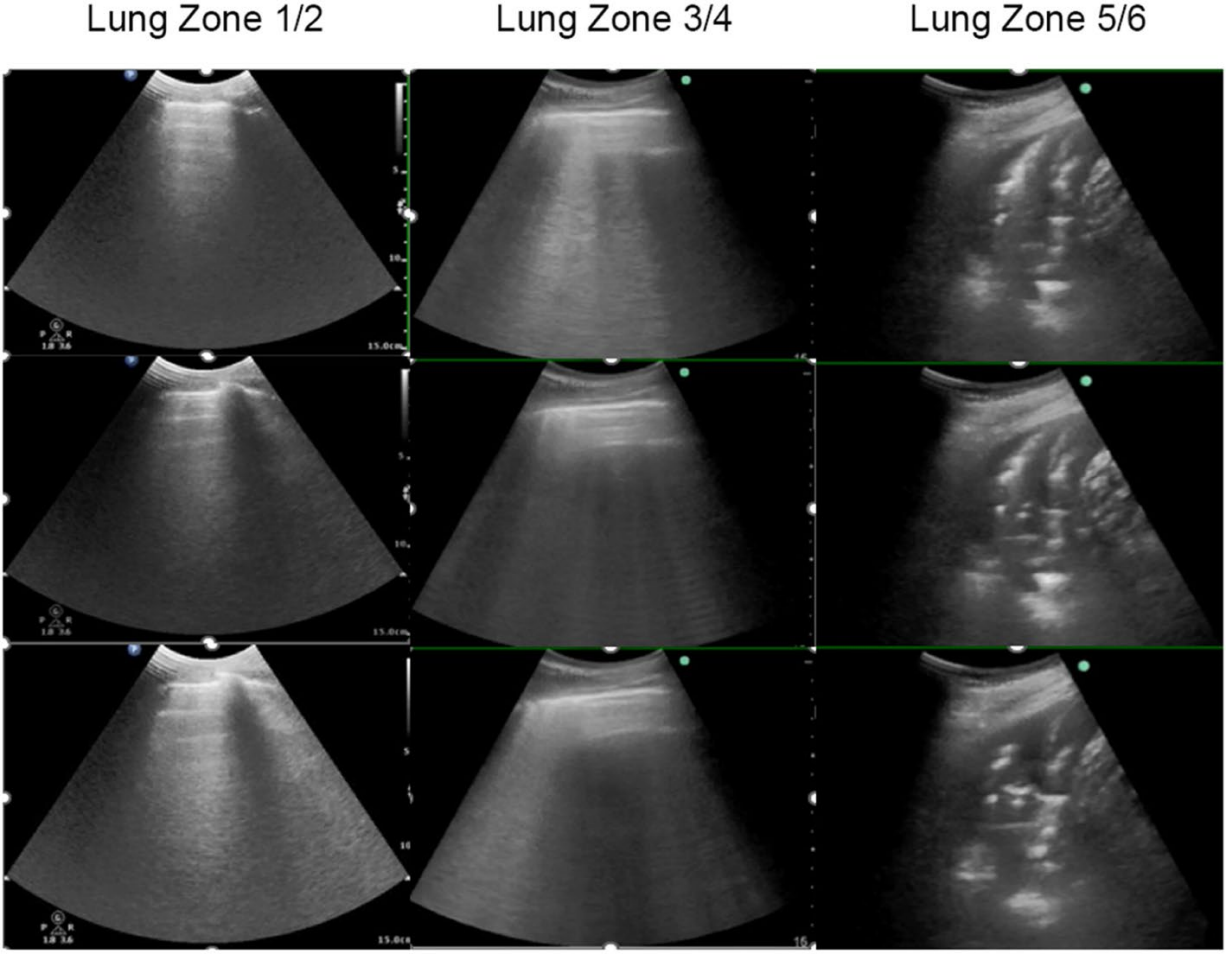
Lung ultrasound on admission

A 15-year-old female, with height of 165 cm and predicted body weight (PBW) of 57 kg, fell from the sixth floor on January 9, 2023. She did not experience any loss of consciousness after falling injury, with a Glasgow Coma Scale (GCS) of 14 (E4V5M5). Subsequently, she was promptly transferred to a local hospital where she received various treatments including intubation, blood transfusion, appendix repair, bilateral closed thoracic drainage, pelvic fracture external fixation, and traction of the right lower limb. Then she was transferred to our surgical intensive care unit (SICU) on January 12, 2023. The patient had a history of suspected depression. On admission, her general condition was as follows (Table 1): temperature of 36.5℃; pulse rate of 132 beats/min; respiratory rate of 18 breaths/min; and blood pressure of 146/92 mmHg (on norepinephrine 0. 4ug/kg/min). She was in deep analgesia, sedation, and muscular relaxation. Both pupils were measured at 2 mm with dull light reflexes. The patient was admitted with mechanical ventilation using both assist-controlled ventilation (A/C) and volume-controlled ventilation (VC) modes with the following settings: tidal volume (VT) of 8.8 ml /kg of PBW; frequency of 14 times/min; fraction of inspired oxygen (FiO2) of 100%; positive end-expiratory pressure (PEEP) of 12cmH2O. The ratio of arterial oxygen partial pressure to fraction of inspired oxygen (P/F ratio) was 70.1 and peripheral oxygen saturation (SPO2) was 95%. She underwent a chest X-ray that showed multiple patchy shadows in both lungs with reduced transmittance (Fig. 1). A chest computerized tomography (CT) scan indicated the presence of pulmonary contusion complicated with infection, and pleural effusion (Fig. 2). The lung ultrasound indicated gravity-dependent distribution of pulmonary fluid (Fig. 3). Based on the findings from the admission examination and previous medical records, an initial diagnosis of falling injury, systemic inflammatory response syndrome, severe acute respiratory distress syndrome, hemorrhagic shock, bilateral hemopneumothorax, and multiple organ dysfunction was established.

**Table 1:** The patient’s vital signs and laboratory data on admission

	Measurement		Result	Reference range/Unit
Vital signs	Body temperature (T)		36.5	℃
	Pulse rate (P)		132	beats/min
	Respiratory rate (R)		18	breath/min
	Blood pressure (BP)		146/92	mmHg
Central nervous system	Richmond Agitation and Sedation Scale (RASS)		-4	-5-+4
	Pupillary size	Left	2	millimeter
		Right	2	
Circulatory system	Central venous pressure (CVP)		5	5–12 cmH2O
	Urine output per hour		30–50	ml/h
	Lactic acid (Lac)		1.4	1.0-1.8 mmol/L
Respiratory system	PaO2/FiO2		70.1	mmHg
	Oxygen Saturation		95	%
Blood/Coagulation system	Hemoglobin		75	115–150 g/L
	Platelet		32	100–300(×10^9^/L)
	Prothrombin time (PT)		13.7	9.6–12.8 s
	Activated Partial Thromboplastin Time (APTT)		20.8	24.8–33.8 s
	Fibrinogen		2.62	2.0–4.0 g/L
	D-Dimer		> 38	< 0.55 mg/I FEU
Digestive system	Occult blood test of gastric juice		+	-
Liver function	Alanine aminotransferase (ALT)		210	< 40 IU/L
	Aspartic aminotransferase (AST)		391	< 35 IU/L
	Albumin (Alb)		24.3	40.0–50.0 g/L
Kidney function	Creatinine		124	21–77 umol/L
	Urea		7.9	2.4–7.2 mmol/L
Inflammatory index	White blood cells		7.52	4.1–11.0(×109/L)
	C-reactive protein (CRP)		109	< 5 mg/L
	Interleukin 6 (IL-6)		4184	0–7.0 pg/ml
	Procalcitonin (PCT)		9.65	< 0.046 ng/ml
Electrolyte	Sodium (Na)		139.1	136–145 mmol/L
	Potassium (K)		3.73	3.5–5.1 mmol/L
	Chloride (CL)		111.0	98–107 mmol/L
Myocardial marker	Brain natriuretic peptide (BNP)		5960	0-334 ng/L
	Myoglobin (Mb)		787.9	< 58.0 ng/ml
	CK-MB		8.77	< 2.88 ng/ml
	TnT		305.7	0–14 ng/L

Initial therapy after admission was as follows: mechanical ventilation in A/C (VC) mode with VT of 7 ml/kg of PBW, frequency of 24 times/min, and FiO2 of 70%; fiberoptic bronchoscopy was performed to clear airway secretions; mesalamine (1.2ug/kg/min) for maintenance of circulation; fentanyl 0.5 mg (2.5ug/kg/h), midazolam 50 mg (0.1 mg/kg/h), and propofol 600 mg (1 mg/kg/h) for analgesia and sedation; vecuronium bromide 20 mg (2.1 ml/h) for muscle relaxant; polyene phosphatidylcholine 20 ml and ilaprazole sodium 10 mg once daily for stress-induced injury; piperacillin sodium and tazobactam sodium 4500 mg every 8 h for anti-infection; external fixation and traction for fractures. Restrictive fluid resuscitation was implemented with the target of mean arterial pressure (MAP) maintained at 70–80 mmHg and continuous gastrointestinal decompression with intra-abdominal pressure (IAP) below 20 mmHg.

Arterial blood gas analysis was repeated 8 h later and her P/F ratio was 95. FiO2 was then adjusted from 70 to 100%, while the rest of the parameters remained unchanged. We performed fiberoptic bronchoscopy several times and took serial arterial blood gas analysis. The patient’s PaO2 fluctuated at 70–90 mmHg, SPO2 fluctuated at 98–99% and P/F ratio was still less than 100. We considered that hypoxemia was associated with gravity-dependent atelectasis, so we performed lung recruitment for her. We took a stepwise increase in PEEP and gradually titrated PEEP to 20 cmH2O, however, her P/F ratio did not improve significantly. We did not further increase PEEP because of the high risk of pneumothorax. Prone positioning was contraindicated due to pelvic fracture and traction.

On day 2, the mesalamine was gradually discontinued as fluid resuscitation progressed. Continuous bedside electrocardiogram (ECG) monitoring revealed that the heart rate fluctuated between 100 and 110 beats/min, systolic blood pressure between 90 and 110 mmHg, and diastolic blood pressure between 60 and 70 mmHg. SPO2 levels were lower compared to previous measurements and fluctuated between 92 and 96%. Intravenous furosemide at a dose of 20 mg was added at a rate of 2 ml/h to alleviate pulmonary fluid overload. The patient exhibited negative fluid balance following diuresis. Subsequent reevaluation of blood gas analysis demonstrated a P/F ratio of 85.5, without significant improvement in SPO2. Furosemide was discontinued due to elevated lactate levels.

Administration of vecuronium bromide and propofol was ceased. The ventilator mode was changed to APRV with initial parameter settings as follows: FiO2 100%; Phigh 27 cmH2O; Plow 5 cmH2O; release frequency of 18 cycles/min; and Tlow was adjusted to achieve termination of peak expiratory flow rate (PEFR) equal to or more than 50% of PEFR. Following the transition to APRV mode, the mean airway pressure increased from 15 to 23 cmH2O, SPO2 gradually improved and reached 100%, and the P/F ratio was 350, indicating an amelioration of hypoxemia. FiO2 was then reduced to 50%, while the P/F ratio was 228.8. The patient’s sputum culture revealed the presence of carbapenem-resistant *Acinetobacter baumannii*. Furthermore, the galactomannan (GM) test conducted on alveolar lavage fluid yielded a positive result, and bronchoscopy revealed minimal pseudomembrane adhering to the mucosa of the left main bronchus. Consequently, the antibiotic regimen was escalated with tigecycline 50 mg every 12 h, meropenem 500 mg every 8 h, and caspofungin 50 mg once daily.

**Fig. 4:**
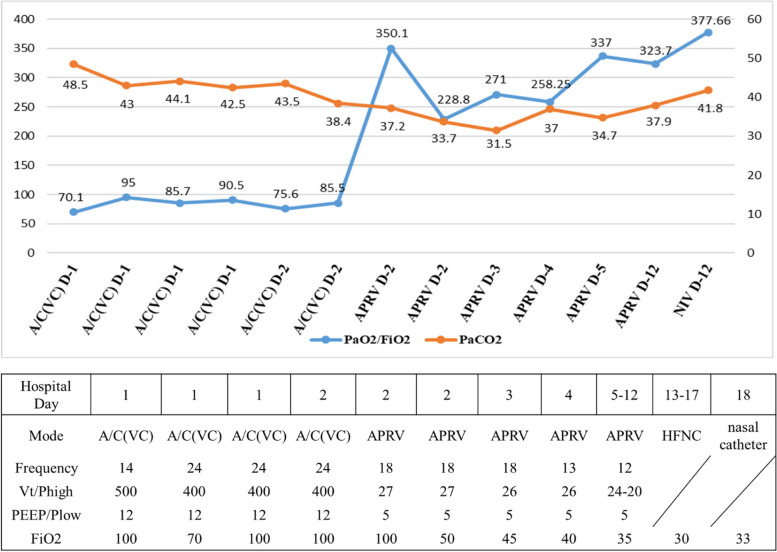
Changes in ventilation settings, PaO2/FiO2 Ratio and PaCO2 after admission

No ventilator asynchrony occurred during APRV mode ventilation. From the third day of admission, a gradual weaning was implemented. The Phigh was reduced by 2 cmH2O, the FiO2 was decreased by 5%, and the release frequency was lowered from 18 to 12 cycles/min. We conducted serial blood gas analysis throughout this period during which the arterial oxygen partial pressure (PaO2) was maintained at 100–150 mmHg, partial pressure of carbon dioxide (PaCO2) at 30–40 mmHg and the P/F ratio at 250–350 (Fig. 4). On day 12, when Phigh was reduced to 20 cmH2O and FiO2 decreased to 35%, she underwent a spontaneous breathing test (SBT) and successfully passed. Subsequently, she was extubated followed by noninvasive ventilation in Spontaneous/Timed (S/T) mode with FiO2 set at 30%, inspiratory positive airway pressure (IPAP) at 14 cmH2O, and expiratory positive airway pressure (EPAP) at 6 cmH2O. The following P/F ratio was 377.66. She was treated with a high-flow nasal cannula (HFNC) the day after extubation.

**Fig. 5:**
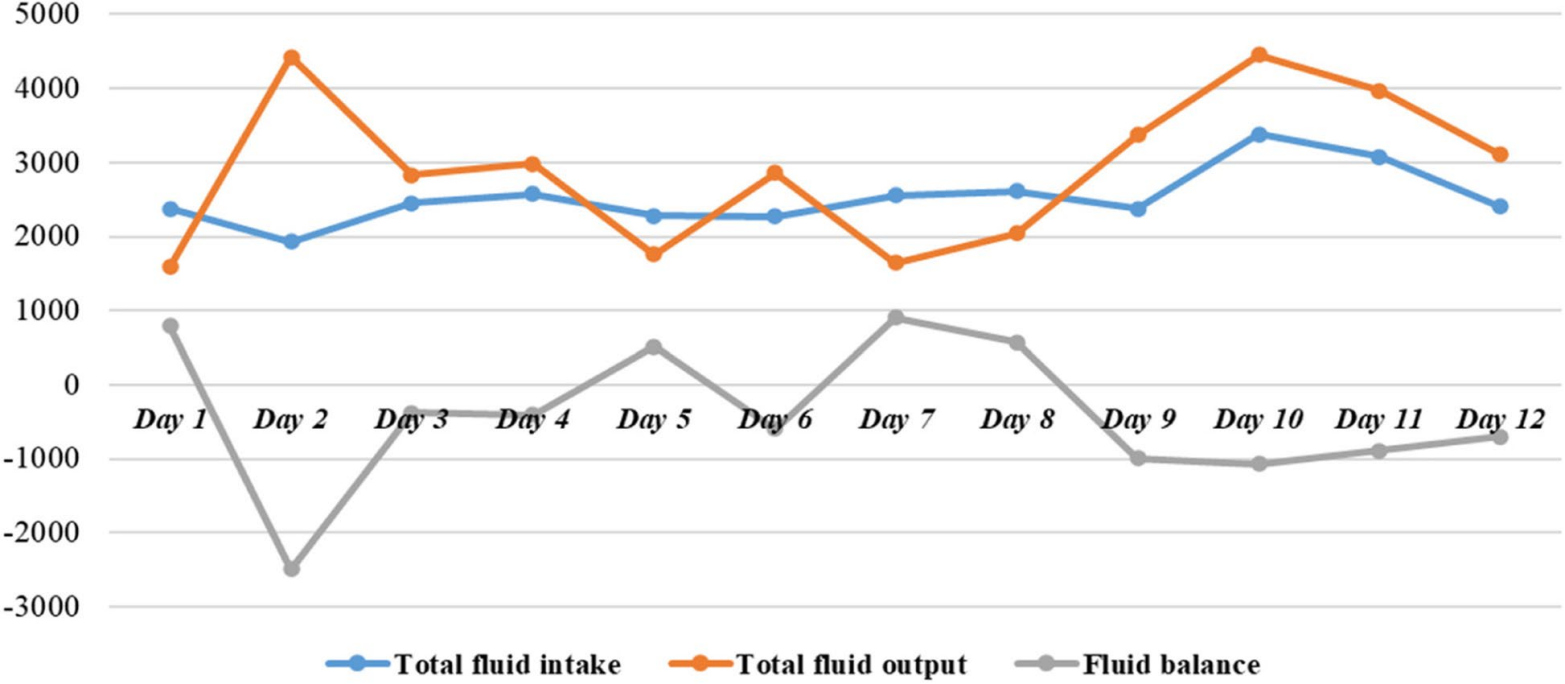
Balance of fluid during invasive mechanical ventilation

**Fig. 6:**
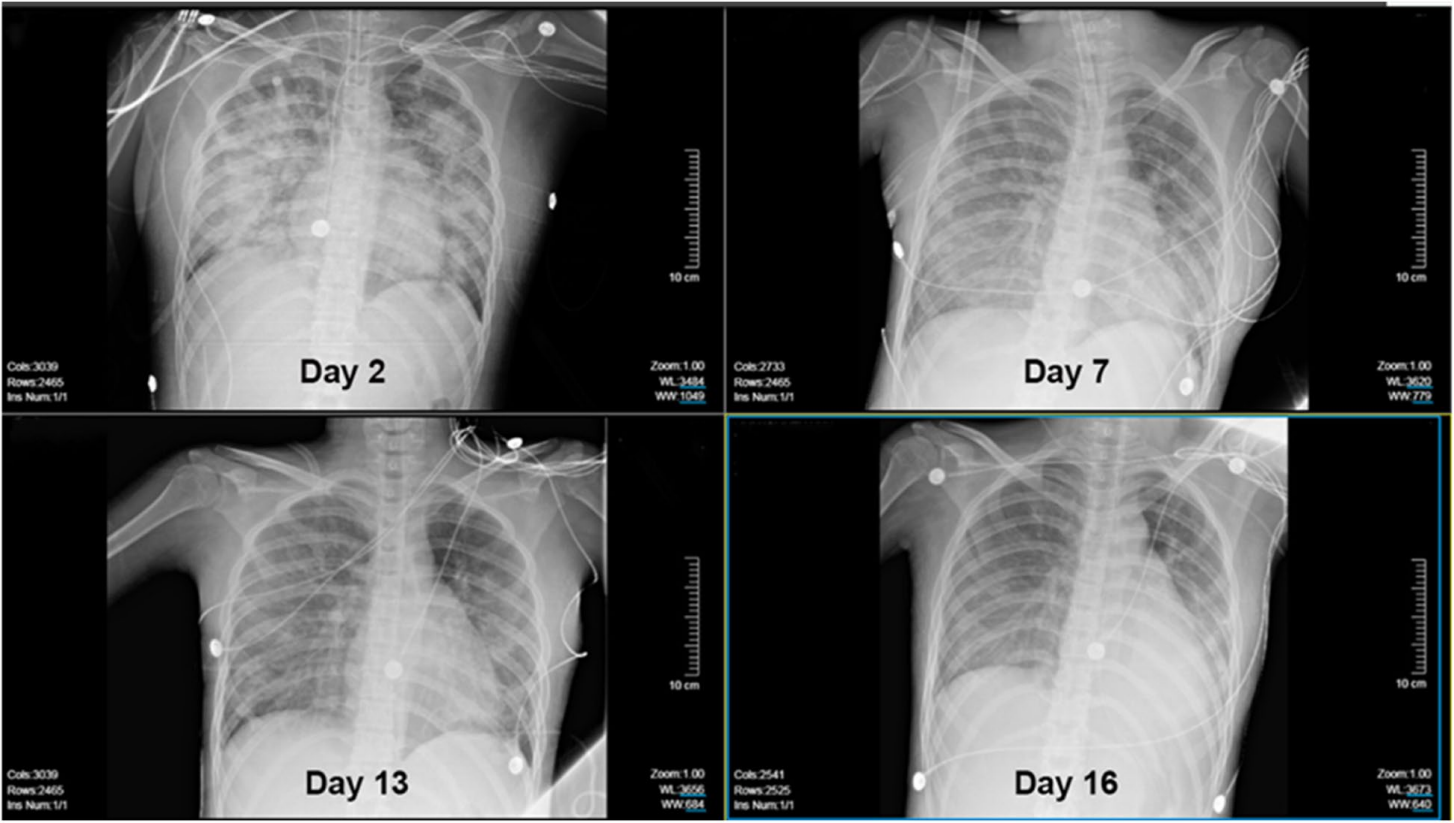
Chest X-ray during hospitalization

**Fig. 7:**
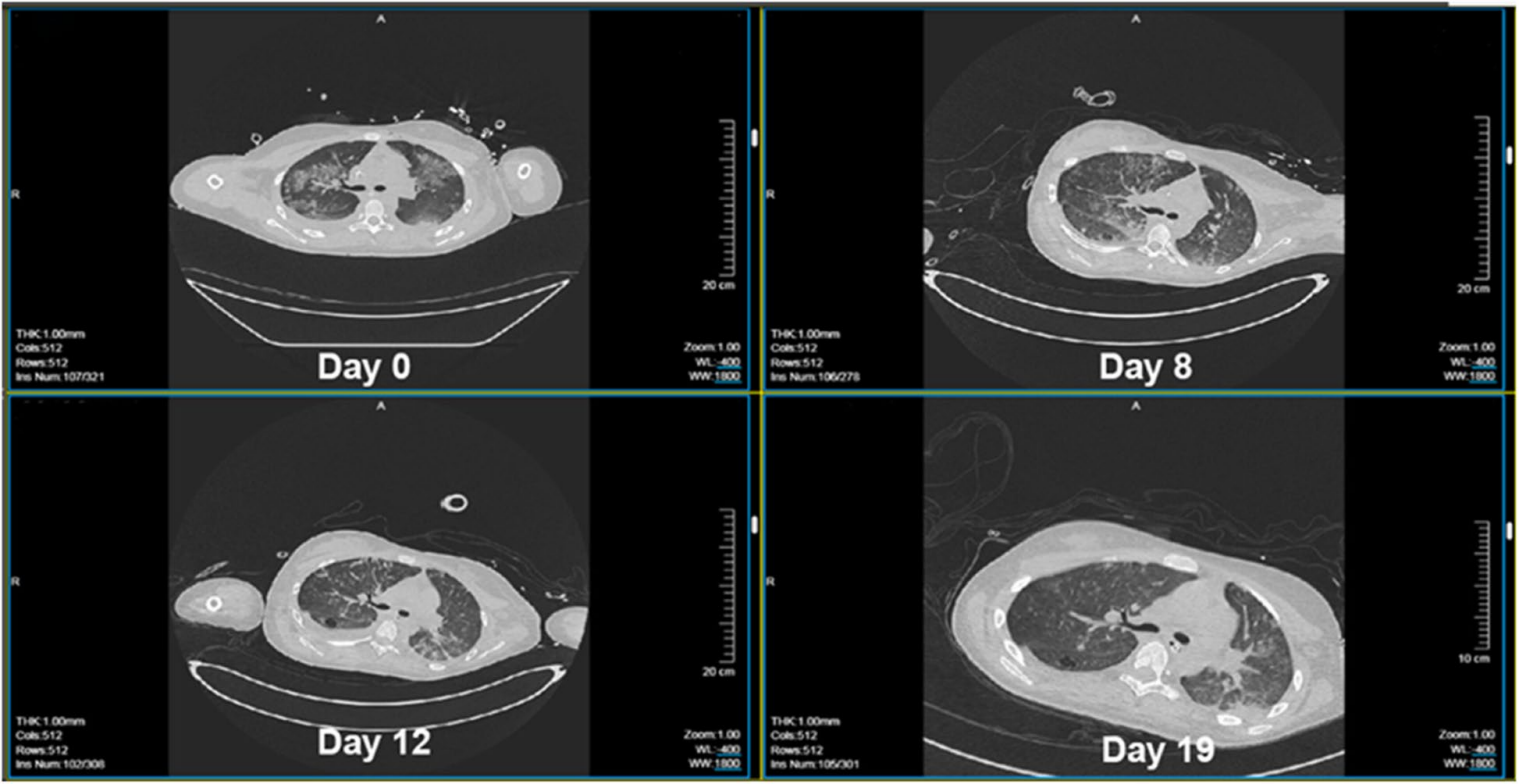
Thoracal computerized tomography scan during hospitalization

During the patient’s hospitalization, we performed daily suction of airway secretions and controlled fluid intake and output (Fig. 5). With the treatments of multiple organ support and protection, control of stress and inflammation as well as anti-infection, the patient’s condition gradually improved. Subsequent chest X-rays and CT scans revealed a progressive improvement in lung imaging (Fig. 6) and gradual resolution of pulmonary infection (Fig. 7). After instituting APRV, there was no episode of air leak. On day 7, the intercostal drainage tubes were removed. On day 17, HFNC was replaced with the nasal catheter. She was transferred from the SICU to the Trauma Medicine Center later for further treatment. She underwent internal fixation of right femoral fracture and pelvis fracture. After 42 days of hospitalization, she was discharged from the hospital and maintained regular follow-up at our outpatient clinic.

## Discussion

ARDS is an acute diffuse, inflammatory lung injury characterized by damage to the pulmonary vascular endothelium and alveolar epithelium with increased permeability, lung edema, and gravity-dependent atelectasis, all of which contribute to loss of aerated lung tissue with increased shunt, and decreased lung compliance [6]. Mechanical ventilation is an essential life support for patients with ARDS, aiming to ensure alveolar ventilation, maintain lung volumes, and improve oxygenation while minimizing lung injury. An in-depth understanding of lung protective ventilation strategies, ventilation modes, PEEP settings, and lung recruitment maneuvers is crucial. When performing mechanical ventilation for patients with ARDS, volume-controlled ventilation or pressure-controlled ventilation is recommended. Current recommendations on lung-protective ventilation include the use of low VT of 4–6 mL/kg of PBW, plateau pressure less than 30 cmH2O, and driving pressure less than 14 cmH2O [7, 8]. Moreover, PEEP should be individualized and set at the lowest level that is needed to attain minimal acceptable SpO2 (88–92%) or PaO2 (55–70 mmHg). Rescue therapies such as prone positioning, recruitment maneuvers, extracorporeal membrane oxygenation (ECMO), and extracorporeal carbon dioxide removal could be considered for patients with severe ARDS [9]. Utilizing low VT and low PEEP can help prevent volutrauma caused by overdistension, but there is a risk of alveolar collapse. Prone positioning ventilation is an effective measure to improve pulmonary heterogeneity and gravity-dependent atelectasis. However, it is contraindicated for patients with severe spine, pelvis, chest wall, or abdominal injuries. The optimal PEEP settings and lung recruitment methods for ARDS are still controversial. For moderate to severe ARDS, high PEEP and recruitment maneuvers may be considered, although they are not routinely recommended due to the potential risks of hemodynamic compromise and ventilator-induced lung injury (VILI) [7–9]. The literature on guiding treatment of mechanically ventilated patient with severe ARDS and trauma is scant. Further, the utility of APRV mode of ventilation is unclear in severe ARDS.

APRV is a time-cycled, pressure-controlled, intermittent mandatory ventilation mode that was first described by Downs and Stock in 1987 [10]. APRV applies two different levels of continuous positive airway pressure (CPAP) with a brief intermittent release phase, allowing unrestricted spontaneous breathing throughout the ventilation cycle [5, 10]. The key settings of APRV include Phigh, Plow, Thigh, and Tlow. Phigh represents the high-level CPAP of the time-triggered mandatory breath, the corresponding time is Thigh. Tlow represents the release time or expiratory time. Plow refers to the low-level CPAP or expiratory pressure [11]. APRV is characterized by extreme inverse inspiratory-expiratory (I: E) ratios that prolong inspiratory time to maintain high airway pressure and shorten expiratory time to allow the release of only partial lung volume, preventing alveolar collapse caused by excessive gas leakage while ensuring gas exchange and carbon dioxide release, finally achieve the purpose of lung recruitment [5]. Using APRV mode does have its downside as its long inspiratory time may induce hypercapnia. More importantly, the degree of hypercapnia and respiratory acidosis tolerated by each patient varies. Continuous monitoring of carbon dioxide during invasive ventilation should be used in patients with ARDS to assess adequacy of ventilation [4].

APRV has the following advantages over conventional mechanical ventilation. APRV generates tidal volume through spontaneous breathing besides the differences between the high-level and the low-level CPAP. The prolonged inspiratory time of APRV increases mean airway pressure without increasing peak airway pressure and reduces significant fluctuations in airway pressure. Therefore, the occurrence of barotrauma and hemodynamic compromise should be less in APRV than in conventional mechanical ventilation [3, 10]. APRV preserves spontaneous breathing and therefore provides additional hemodynamic benefits by decreasing intrathoracic pressure and increasing venous return through diaphragmatic movement. It also reduces patient-ventilator asynchrony, improves comfort, reduces sedation and muscle relaxant requirements, and thus reduces the risk of ventilator-associated pneumonia [11, 12].

Despite these advantages, there is still a lack of strong evidence to support the superiority of APRV over conventional mechanical ventilation. Zhou et al. conducted an RCT comparing the APRV with the low tidal volume (LTV) in adult ARDS patients. Compared with LTV, early application of APRV improved oxygenation and respiratory system compliance, decreased plateau airway pressure, and reduced the duration of mechanical ventilation and ICU stay. The mortality rate and hospital length of stay in the two groups were similar [13]. An RCT conducted by Lalgudi et al. examined the effects of APRV and LTV in children with ARDS. The study found that patients in the APRV group demonstrated an earlier improvement in oxygenation compared to those in the LTV group. However, the trial was prematurely terminated due to higher mortality rates observed in the APRV group as opposed to the LTV group [14]. Two studies conducted on trauma populations showed that both APRV and LTV exhibited similar safety profiles, but APRV may lead to prolonged mechanical ventilation [15, 16]. Possible explanations for the discrepancies in results include the limited sample size of available studies, imbalanced baselines that patients in the APRV group often had more severe conditions, and variations in parameter settings and weaning protocols for APRV. In addition, the heterogeneity of the study population and the timing of APRV application may have influenced the outcomes. And trauma-related ARDS may differ pathophysiologically from non-trauma-related ARDS such as sepsis [17]. Factors such as the severity of thoracic trauma, degree of hemorrhagic shock, and volume of red blood cell infusion are associated with an early-onset ARDS after trauma [18, 19]. A systematic review focusing on observational studies of trauma-related ARDS suggested that early implementation of APRV may reduce mortality in this specific population [20]. Furthermore, for patients with moderate to severe ARDS, early application of APRV can improve ventilation-perfusion ratio and lung heterogeneity [21].

In this case, the patient was admitted with severe ARDS after trauma. The LTV strategy was usually used with deep analgesia and sedation as well as muscle relaxants. Incremental PEEP titration to open the collapsed alveoli was also employed. But her P/F ratio was consistently less than 100. Upon implementing APRV, a notable improvement in oxygenation was observed without any detrimental effects on circulation. We speculate that APRV reopened the collapsed alveoli of gravity-dependent zones and maintained alveolar recruitment, thereby improving the ventilation-perfusion ratio and interrupting the vicious cycle of hypoxemia. The successful application of APRV prevented us from taking further treatments such as ECMO that may exacerbate the pathophysiologic disorders. With the combination treatment of analgesia, sedation, anti-infection, fluid and airway management, the patient gradually recovered. Nevertheless, it should be noted that while APRV proved effective in this trauma-related ARDS case, APRV should be used with caution in patients with lung injury, multiple rib fractures, thoracic instability, or high intracranial pressure. The high average airway pressure in APRV may increase the risk of barotrauma or even lead to pneumothorax. Although spontaneous breathing can bring a series of physiological benefits, patients are at risk of developing VILI when alterations occur in breathing mechanics and respiratory effort, which may lead to enhanced voluntary breathing, marked fluctuations in tidal volume and transpulmonary pressure. Transpulmonary pressure monitoring using oesophageal manometry may be helpful to understand the lung compliance, patient effort and to manage protective ventilation [22]. Despite the presence of bilateral hemopneumothorax, the therapeutic benefits of APRV manifested before any adverse effects. During the administration of APRV, we meticulously monitored and regulated airway pressure, so the patient did not develop VILI. Furthermore, the patient had no significant rib fractures and bilateral closed thoracic drainage tubes had been previously placed at a local hospital. So APRV was relatively safe in this patient.

APRV offers an alternative approach to maximize lung recruitment and oxygenation, but present clinical trials have not demonstrated the survival benefit of APRV over conventional ventilation strategies. Moreover, protocols for initiation, titration, and weaning methods of APRV are limited. High-quality RCTs are still needed to explore the safety and efficacy of APRV in patients with ARDS, especially in children [13–15, 23–26]. We hope this case report presents a potential option for trauma-related ARDS and serves as a basis for future research.

## Conclusion

APRV maximizes and maintains alveolar ventilation, preserves spontaneous breathing, takes advantage of diaphragmatic function, improves ventilation-perfusion ratio, and features lung-protective recruitment. APRV may be considered early in the management of trauma patients with severe ARDS when prone position ventilation cannot be performed and refractory hypoxemia persists despite conventional ventilation and lung recruitment maneuvers. When using APRV, the tolerance of the patient should be evaluated and the parameters of APRV should be set according to actual conditions.

## Data Availability

Data sharing is not applicable to this article as no datasets were generated or analysed.

## Abbreviations

A/C: Assist-controlled ventilation
ARDS: Acute respiratory distress syndrome
APRV: Airway pressure release ventilation
CPAP: Continuous positive airway pressure
ECG: Electrocardiogram
ECMO: Extracorporeal membrane oxygenation
EPAP: Expiratory positive airway pressure
FiO2: Fraction of inspired oxygen
HFNC: High-flow nasal cannula
IAP: Intra-abdominal pressure
IPAP: Inspiratory positive airway pressure
LTV: Low tidal volume
MAP: Mean arterial pressure
PaO2: Arterial oxygen partial pressure
PaCO2: Partial pressure of carbon dioxide
PBW: Predicted body weight
PEEP: Positive end-expiratory pressure
PEFR: Peak expiratory flow rate
P/F Ratio: Ratio of arterial oxygen partial pressure to fraction of inspired oxygen
SBT: Spontaneous breathing test
SICU: Surgical intensive care unit
SPO2: Peripheral oxygen saturation
S/T: Spontaneous/Timed
VC: Volume-controlled ventilation
VILI: Ventilator-induced lung injury
VT: Tidal volume

## References

[1] Bellani G, Laffey JG, Pham T, Fan E, Brochard L, Esteban A, Gattinoni L, van Haren F, Larsson A, McAuley DF (2016). Epidemiology, patterns of Care, and mortality for patients with Acute Respiratory Distress Syndrome in Intensive Care Units in 50 countries. JAMA.

[2] Derdak S. Acute respiratory distress syndrome in trauma patients. J Trauma. 2007;62(6 Suppl):S58. 10.1097/TA.0b013e318065ab4e. 10.1097/TA.0b013e318065ab4e 17556976

[3] Ramin S, Charbit J, Jaber S, Capdevila X (2019). Acute respiratory distress syndrome after chest trauma: Epidemiology, specific physiopathology and ventilation strategies. Anaesth Crit Care Pain Med.

[4] Emeriaud G, Lopez-Fernandez YM, Iyer NP, Bembea MM, Agulnik A, Barbaro RP, Baudin F, Bhalla A, Brunow de Carvalho W, Carroll CL (2023). Executive summary of the Second International guidelines for the diagnosis and management of Pediatric Acute respiratory distress syndrome (PALICC-2). Pediatr Crit Care Med.

[5] Mallory P, Cheifetz I (2020). A comprehensive review of the use and understanding of airway pressure release ventilation. Expert Rev Respir Med.

[6] Matthay MA, Arabi Y, Arroliga AC, Bernard G, Bersten AD, Brochard LJ, Calfee CS, Combes A, Daniel BM, Ferguson ND (2023). A New Global Definition of Acute Respiratory Distress Syndrome. Am J Respir Crit Care Med.

[7] Battaglini D, Fazzini B, Silva PL, Cruz FF, Ball L, Robba C, Rocco PRM, Pelosi P. Challenges in ARDS Definition, Management, and identification of effective personalized therapies. J Clin Med. 2023;12(4). 10.3390/jcm12041381. 10.3390/jcm12041381 PMC9967510 36835919

[8] Banavasi H, Nguyen P, Osman H, Soubani AO (2021). Management of ARDS - what works and what does not. Am J Med Sci.

[9] Battaglini D, Sottano M, Ball L, Robba C, Rocco PRM, Pelosi P (2021). Ten golden rules for individualized mechanical ventilation in acute respiratory distress syndrome. J Intensive Med.

[10] Downs JB, Stock MC (1987). Airway pressure release ventilation: a new concept in ventilatory support. Crit Care Med.

[11] Daoud EG, Farag HL, Chatburn RL (2012). Airway pressure release ventilation: what do we know?. Respir Care.

[12] Walkey AJ, Nair S, Papadopoulos S, Agarwal S, Reardon CC (2011). Use of airway pressure release ventilation is associated with a reduced incidence of ventilator-associated pneumonia in patients with pulmonary contusion. J Trauma.

[13] Zhou Y, Jin X, Lv Y, Wang P, Yang Y, Liang G, Wang B, Kang Y (2017). Early application of airway pressure release ventilation may reduce the duration of mechanical ventilation in acute respiratory distress syndrome. Intensive Care Med.

[14] Lalgudi Ganesan S, Jayashree M, Chandra Singhi S, Bansal A (2018). Airway Pressure Release Ventilation in Pediatric Acute Respiratory Distress Syndrome. A Randomized Controlled Trial. Am J Respir Crit Care Med.

[15] Maung AA, Schuster KM, Kaplan LJ, Ditillo MF, Piper GL, Maerz LL, Lui FY, Johnson DC, Davis KA (2012). Compared to conventional ventilation, airway pressure release ventilation may increase ventilator days in trauma patients. J Trauma Acute Care Surg.

[16] Maxwell RA, Green JM, Waldrop J, Dart BW, Smith PW, Brooks D, Lewis PL, Barker DE (2010). A randomized prospective trial of airway pressure release ventilation and low tidal volume ventilation in adult trauma patients with acute respiratory failure. J Trauma.

[17] Calfee CS, Eisner MD, Ware LB, Thompson BT, Parsons PE, Wheeler AP, Korpak A, Matthay MA, Acute Respiratory Distress Syndrome Network NHL, Blood I. Trauma-associated lung injury differs clinically and biologically from acute lung injury due to other clinical disorders. Crit Care Med. 2007;35(10):2243–50. 10.1097/01.ccm.0000280434.33451.87. 10.1097/01.ccm.0000280434.33451.87 PMC2765812 17944012

[18] Reilly JP, Bellamy S, Shashaty MG, Gallop R, Meyer NJ, Lanken PN, Kaplan S, Holena DN, May AK, Ware LB (2014). Heterogeneous phenotypes of acute respiratory distress syndrome after major trauma. Ann Am Thorac Soc.

[19] Gong MN, Thompson BT, Williams P, Pothier L, Boyce PD, Christiani DC (2005). Clinical predictors of and mortality in acute respiratory distress syndrome: potential role of red cell transfusion. Crit Care Med.

[20] Andrews PL, Shiber JR, Jaruga-Killeen E, Roy S, Sadowitz B, O’Toole RV, Gatto LA, Nieman GF, Scalea T, Habashi NM (2013). Early application of airway pressure release ventilation may reduce mortality in high-risk trauma patients: a systematic review of observational trauma ARDS literature. J Trauma Acute Care Surg.

[21] Li R, Wu Y, Zhang H, Wang A, Zhao X, Yuan S, Yang L, Zou X, Shang Y, Zhao Z (2023). Effects of airway pressure release ventilation on lung physiology assessed by electrical impedance tomography in patients with early moderate-to-severe ARDS. Crit Care.

[22] Vedrenne-Cloquet M, Khirani S, Khemani R, Lesage F, Oualha M, Renolleau S, Chiumello D, Demoule A, Fauroux B (2023). Pleural and transpulmonary pressures to tailor protective ventilation in children. Thorax.

[23] Cheng J, Ma A, Dong M, Zhou Y, Wang B, Xue Y, Wang P, Yang J, Kang Y (2022). Does airway pressure release ventilation offer new hope for treating acute respiratory distress syndrome?. J Intensive Med.

[24] Chen C, Zhen J, Gong S, Yan J, Li L (2021). Efficacy of airway pressure release ventilation for acute respiratory distress syndrome: a systematic review with meta-analysis. Ann Palliat Med.

[25] Carsetti A, Damiani E, Domizi R, Scorcella C, Pantanetti S, Falcetta S, Donati A, Adrario E (2019). Airway pressure release ventilation during acute hypoxemic respiratory failure: a systematic review and meta-analysis of randomized controlled trials. Ann Intensive Care.

[26] Sklar MC, Patel BK, Beitler JR, Piraino T, Goligher EC (2019). Optimal ventilator strategies in Acute Respiratory Distress Syndrome. Semin Respir Crit Care Med.

